# Development and Validation of Sleep Disturbance Questionnaire in Patients with Acute Coronary Syndrome

**DOI:** 10.1155/2014/978580

**Published:** 2014-10-22

**Authors:** Elham Sepahvand, Rostam Jalali, Behnam Khaledi Paveh, Mansour Rezaei

**Affiliations:** ^1^Faculty of Nursing and Midwifery, Kermanshah University of Medical Sciences, Kermanshah 67198-51351, Iran; ^2^Research Center of Social Development and Health Promotion, Kermanshah University of Medical Sciences, Kermanshah 67198-51351, Iran; ^3^Sleep Disorder Research Center, Kermanshah University of Medical Sciences, Kermanshah 67198-51351, Iran; ^4^Faculty of Medicine, Kermanshah University of Medical Sciences, Kermanshah 67198-51351, Iran

## Abstract

*Background and Objectives*. Severe sleep disturbance is a common problem among patients in cardiac care units (CCUs). There are questionnaires to measure sleep disturbances. Therefore, the present study seeks to design a valid and reliable questionnaire to assess sleep disturbance in patients with acute coronary syndrome (ACS) hospitalized in CCUs.* Materials and Methods*. In the present methodological research, items of the questionnaire were extracted through a systematic review. The validity and reliability of the questionnaires was assessed by face validity, content validity, construct validity, Cronbach's alpha coefficient, and test-retest methods.* Results*. Factor analysis provided a questionnaire of 23 items on 5 dimensions of sleep disturbance in coronary patients: “sleep onset and continuity disorder,” “disorder in daytime functioning,” “sleep disturbance caused by environmental factors,” “sleep disturbance as a result of cardiac diseases,” and “respiratory disorders during sleep.” Furthermore, test-retest analysis showed a reliability correlation coefficient of *r* = 0.766 and *α* Cronbach's reliability (*α* = 0.855).* Conclusion*. Sleep disturbance questionnaire for patients with ACS hospitalized in coronary care unit (CCU) was identified in 5 dimensions and assessed for validity and reliability. To control and improve the sleep quality of CCU hospitalized patients, we need to identify and remove predisposing factors.

## 1. Introduction

Sleep is one of the basic human needs and a reversible mode of behavior which is associated with changes in consciousness and unresponsiveness to environmental stimulus [1–4]. Sleep in fact provides time for body system to modify, restructure, and repair itself for the coming course [1, 5]. Repair, reorganization, memory enhancement, and learning take place during sleep [1]. All individuals spend about a third of their life sleeping [6].

Regulating the circle of sleep and wake is a complicated process. It is controlled by creating a balance between homeostatic need to sleep, circadian rhythm, and interaction among neural mediators [7].

Previous studies have indicated that sleep is a main and basic need for recovery and survival of patients in CCUs [1, 8]. Sleep also plays an important role in cardiovascular function hence sleep deprivation causes severe anxiety, irritability, and increased oxygen demand of the heart muscle [1]. Although critically ill patients hospitalized in intensive care unit (ICU) need more restful sleep, they are usually exposed to a higher risk of sleep deprivation and poor-quality sleep [9]. Patients with cardiac disease, particularly those who are hospitalized in coronary care unit (CCU), have sleep disturbance which might cause physiological changes during sleep and accordingly negative effects on patient's health [10]. On the other side, insomnia increases the power and speed of contraction of heart muscle and thus heart need for oxygen [1]. Patients hospitalized in CCU have a lower sleep quality compared to the time when they stay at home. Sleep disturbance causes release of epinephrine and norepinephrine and increases the activity of sympathetic system, heart rate, respiratory rate, and incidence of dysrhythmia which are regarded as the intensifying factors of ischemia and infarction of the heart and eventually heart attack [1].

To better understand the relationship between sleep disturbance and its negative consequences on patients, simple and valid questionnaires have been developed [11]. These instruments include objective and subjective devices to measure the quality of sleep. Polysomnography, which requires special equipment and facilities, is a golden standard for measuring sleep and wake patterns and sleep disturbances [12]. Although polysomnography is a credible objective questionnaire which provides information on the quantity of sleep, it is incapable of providing a proper definition of sleep quality [13]. Moreover, applying polysomnography on hospitalized patients is difficult [14].

In addition, another device, actigraphy, is capable of estimating sleep and wake times only based on strong connection between sleep-wake situation and motional activities [12]. Sleep is a subjective experience and the components of sleep quality as well as their importance are different in each individual. Self-report questionnaires are more practical than the other devices in studying sleep disturbances and hence are widely used to study the sleep and wake function. Nurses are therefore concerned to study both the sleep disturbances reported by a patient and their related assessment [12, 13]. Although there are several questionnaires to study sleep disturbances, they are not specific [15].

Pittsburgh sleep quality inventory (PSQI) covers not only several dimensions of sleep disturbance,but also items that might not be related to coronary symptoms [15]. Furthermore, Epworth Sleepiness Scale (ESS) includes 8 items that measure sleepiness in various situations. Failing to cover the necessary items for measuring specific areas is considered as a problem for these questionnaires; however, their main problem still is that they are limited to a certain range of contents. Many of sleep disturbance assessment questionnaires (like Pittsburgh and Epworth) are multidimensional and assess nocturnal sleep problems and daytime fatigue on a general scale [16, 17].

Due to pain, severe underlying disease, and stressful environment of CCU, patients hospitalized in CCU suffer sleep pattern disorder in a form of frequent waking and sleep time reduction followed by sleep quality reduction and worsening of cardiac problems. Therefore, the nurses' main responsibilities are to ensure that patients sleep and get adequate rest. Since Pittsburgh Quality Scale studies sleep disturbance for a period of past month, it is not appropriate to be applied on patients with ACS hospitalized in CCU whose sleep disturbance starts just after their admission in the ward. This fact illustrates another shortcoming of current questionnaires and also highlights the necessity of developing specific and precise questionnaires to assess sleep disturbance in ACS patients. The present study seeks to develop and validate an instrument for sleep disturbance assessments among ACS patients.

## 2. Materials and Methods

The present study follows a methodological research method and is conducted in two stages: (a) defining and developing appropriate items for an ACS-specific questionnaire for sleep disturbance assessment; (b) evaluating the validity and reliability of the questionnaire. As the first step of developing a questionnaire, the relevant items extracted from the conventional questionnaires and sleep assessment tools were reviewed. This was done by performing a comprehensive search in scientific databases like CINAHL, MEDLINE, PubMed, ScienceDirect, Elsevier, and Ovid. The keywords included “Sleep,” “Sleep Disturbance,” “Psychometric,” “Questionnaire,” and “Acute Coronary Syndrome.” Furthermore, the extracted conventional questionnaires used in the assessments of sleep quality and disturbances were specifically and generally translated and their items were added to the items obtained through the search. Similar and duplicated items were either excluded or merged in the final version to form the original questionnaire. Content, face, construct, and concurrent validity were assessed to determine the validity of the questionnaire. To determine content validity of this improved questionnaire, the Content Validity Index (CVI) of each item was separately defined. Finally, only those items whose CVIs for relevance were above 0.75 were kept for further analyses.

In order to measure content validity, the questionnaire was given to experts (4 cardiologists, 3 psychiatrists, 8 members of the faculty of nursing, and 3 nurses in coronary care unit). Their judgments were based on CVI, and each item was rated from 1 to 4 according to its level of relevance, clarity, and simplicity. Then, the total score was divided by probable total score. Following the amendments, the questionnaire was given to the 8 members of the faculty of nursing to score the items based on Waltz and Bausell's index. At this stage, the percentage obtained from Waltz and Bausell's CVI for each item was separately calculated and the items with relevance scores above 0.9 were retained and the rest was excluded (below 90%).

**Figure 1:**
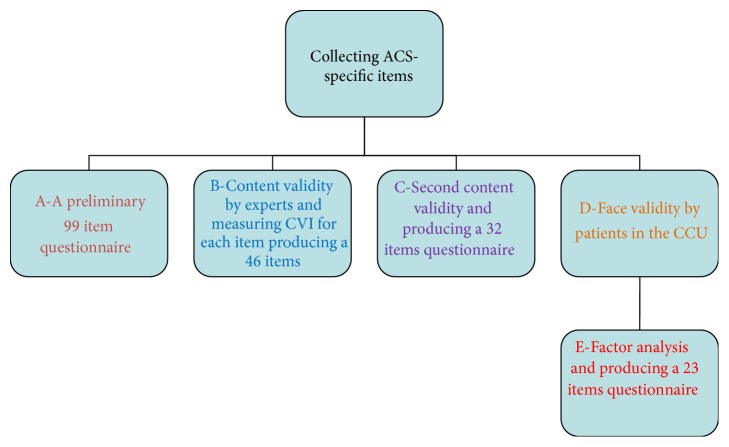
Process of questionnaire development.

The next step was the assessment of face validity. Having made grammatical and appearance adjustments and reviewed the overall structure of the questionnaire, it was distributed among 20 patients in the CCUs to assess the simplicity and intelligibility of the items. Then, necessary modifications were made based on the obtained feedbacks (Figure 1).

The statistical software of SPSS (version 16) was used for construct validity, factorial analysis, internal consistency, and test-retest assessments.

In this study, exploratory factor analysis based on Nunnally and Bernstein's (1994) procedure is applied [18] to determine construct validity with the purpose of examining factor structure of the questionnaire. 221 patients with ACS hospitalized in CCU (two major educational hospitals in Kermanshah, west of Iran) were randomly selected to complete the questionnaire and implement the factor analysis. Only those patients who had been hospitalized for at least two days in CCU and accepted to participate in the study were enrolled. Others including patients who rejected participation or the ones who had a comorbid disease or received opiates were excluded. Moreover, items with a correlation coefficient below 0.40 were excluded. As for the reliability of sleep disturbance assessment questionnaire for coronary patients, Cronbach's alpha coefficient was applied to assess internal consistency and test-retest to assess the stability. The study was funded and approved by the Ethics Committee of Kermanshah University of Medical Sciences.

## 3. Results

Having used multiple CVI, the content validity of the 99 items of the questionnaire was assessed and finally the total number decreased to 32 items. This questionnaire was used for next stages of the study (Figure 1).

In order to increase face validity, the punctuation rules were strictly followed based on the feedbacks of coronary experts and patients. Patients' opinions about the questionnaire were also taken into consideration and unintelligible and unfamiliar items were amended.

**Figure 2:**
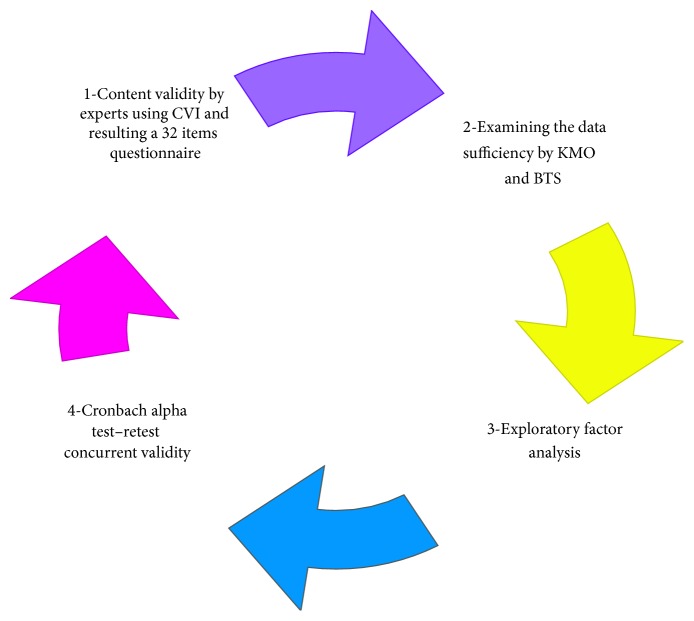
Statistical methods to questionnaire validation.

In order to obtain a consistent set of items for sleep disturbance assessment questionnaire before factor analysis, internal consistency of 32 items of disturbance assessment questionnaire were examined. Then items with a correlation coefficient below 40% were excluded resulting in a 23-item questionnaire. Item weighting of the questionnaire was estimated by Cronbach's alpha (Table 1). Factor analysis was conducted on the remaining 23 items. Before factor analysis, data sufficiency was examined by Kaiser-Meyer-Olkin (KMO) and Bartlett's test of sphericity (BTS). The result of factor analysis indicated KMO of 0.843. Furthermore, the result of BTS indicated *P* ≤ 0.001. After conducting the test by the use of factor analysis, the result indicated 5 dimensions of sleep disturbance in patients with ACSs (Figure 2). Approximately 58% of the total variance of sleep disturbance assessment questionnaire in ACS patients was explained by these 5 factors. The extracted factors from orthogonal rotation were then rotated by varimax rotation to specify the underlying dimensions of sleep disturbance in ACS patients. 5 factors were estimated (Table 2). Dimensions of the questionnaire were named in terms of the items of each dimension. The first dimension which was named sleep onset and continuity disorder consisted of 6 items and represented approximately 24% of the total variance of the questionnaire (Table 3).

Furthermore, the questionnaire's reliability assessments indicated Cronbach's alpha internal consistency coefficient of *r* = 0.855. Correlation coefficient of test-retest indicated *r* = 0.766.

Scoring was based on Likert scale. The points of the scale were as follows: “strongly disagree = 1,” “disagree = 2,” “neither agree nor disagree = 3,” “agree = 4,” and “strongly agree = 5.” The total scores lower than 55 indicate that the patient does not suffer sleep disturbances, and the scores between “56–75,” “76–95,” and “96–115” indicate mild, moderate, and severe sleep disturbances, respectively.

## 4. Discussion

The result of the study indicated a questionnaire in 5 dimensions including: sleep onset and continuity disorder, disorder in daytime functioning, sleep disturbance because of environmental factors, sleep disturbances as a result of cardiac diseases, and respiratory disorders during sleep. The first dimension of sleep disturbance in ACS patients included sleep onset and continuity disorder. Most of the patients reported sleep onset and continuity disorder. It seems that cardiac patients clearly experience sleep problems, as argued by many other studies [8, 19–31].

Sleep onset and continuity disorder and waking earlier than the desired time cause lack of effective sleep and reduction of nocturnal sleep quality in ACS patients. Furthermore, difficulty in falling asleep (insomnia) and frequent waking during the night were found in most of ACS. Therefore, it seems logical to argue that the sleep conditions of ACS patients can not be improved unless these disturbing factors are successfully removed.

The second dimension of the sleep disturbance questionnaire in patients with ACS was “disorder in daytime functioning.” In similar previous studies, the same issues have been addressed. These studies indicate a close relationship between cardiac diseases and sleep problems [20, 21, 32–41].

Revealing the negative factors which disturb CCU hospitalized patients' sleep patterns is an important step toward providing better sleep conditions for them. Although restless leg syndrome (RLS) is assumed as a common sleep disturbance, it is rarely diagnosed. Further studies are necessary to diagnose and control RLS to reduce the consequences of sleep disturbances among CCU hospitalized patients.

Diagnosis, examination, treatment, and assessment of disordered sleep and fatigue are the responsibilities of healthcare personnel. Within a health care team, nurses have a significant role in identifying patients with sleep and daytime functioning disorder and accordingly providing them with better conditions to resolve their disorders. Diagnosis and treatment of sleep disturbance and disorders such as fatigue, malaise, anxiety, and depression in hospitalized patients can help their recovery.

The third dimension of sleep disturbance questionnaire was sleep caused by environmental factors. The environmental factors like CCU environment are some of the most critical causes of sleep disturbance in coronary patients hospitalized in CCU. According to some studies, environmental factors such as noise of devices, light, unusual odors, and medical care interventions are the main causes of sleep disturbance, albeit noise of devices and the routine conversations of the medical personnel were counted as the worst factor [14, 42–46].

It seems that the main factor that causes insomnia in patients is the environment of the CCU. Noise is one of the most critical environmental factors for sleep disturbance. Noise causes increased sympathetic system activities, increased heart rate and blood pressure, difficulty falling asleep, frequent waking, and disturbance in normal sleep pattern. In addition to noise, other factors such as light, pain, drugs, and care and treatment interventions can cause sleep disturbance in patients. Therefore, CCU doctors and nurses should upgrade their knowledge regarding sleep disturbance in patients with critical conditions and its causing factors, as well as the effects of such disorders on patients' health.

The fourth dimension of sleep disturbance questionnaire for patients with ACS is disorder caused by cardiac disease. ACS causes sleep disturbance in patients. Some studies reported that pain and the chronic nature of the disease in patients hospitalized in CCU cause disorders in sleep pattern [14, 43, 45, 47–51].

Patients with ACS experience changes in sleep structure, difficulty in sleep onset and continuity, frequent waking, reduced total sleep duration, and reduced duration of rapid eye movement (REM), and non-REM stages of sleep. The severity of the disease is a critical cause of sleep disturbance that can affect not only the quality and quantity of sleep but also patients' quality of life. REM sleep deprivation leads to REM sleep recurrence phenomenon followed by increased heart rate, hypoxia, cardiac arrhythmias, and hemodynamic instability.

The fifth dimension of sleep disturbance questionnaire for ACS patients included respiratory disorder during sleep. Findings of the studies conducted by researchers showed that obstructive sleep apnea (OSA) was a common disorder among ACS patients [52–57].

Respiratory disorder during sleep in patients with ACS is highly prevalent and patients with respiratory disorders report frequent daytime drowsiness. This questionnaire efficiently reflects these dysfunctions and also stresses the need to raise awareness for early diagnosis and proper care and treatment measures for coronary patients who suffer respiratory disorders during their sleeptime.

## 5. Conclusion

In this study, an improved sleep disturbance questionnaire with 5 dimensions for patients with ACS hospitalized in CCUs was designed and its efficiency was evaluated. It seems that disturbing factors need to be identified and removed in order to improve sleep disturbance among these patients. Although this 23-item questionnaire indicates an appropriate level of validity and reliability, other items can still be added or altered according to the patients' cultural background and special environment of CCUs. Furthermore, this study is the first step for developing an ACS patients-specific questionnaire and the developed questionnaire is still in its early stage.

## Figures and Tables

**Table 1:** Cronbach's alpha if item deleted and item weighting.

Number	Cronbach's alpha if item deleted	Item weighting
1	.797	0.807
2	.795	0.762
3	.794	0.713
4	.807	0.808
5	.803	0.526
6	.790	0.608
7	.795	0.604
8	.793	0.405
9	.804	0.451
10	.798	0.418
11	.797	0.756
12	.793	0.784
13	.803	0.696
14	.798	0.690
15	.795	0.574
16	.802	0.666
17	.800	0.676
18	.798	0.714
19	.800	0.432
20	.796	0.538
21	.792	0.765
22	.796	0.600
23	.810	0.838

**Table 2:** Results of the factorial analysis of rotated component matrix.

Question number	Component				
	1	2	3	4	5
1	.807	.209	.003	.061	−.016
2	.762	.250	−.010	.040	.088
3	.096	.713	.148	−.107	.160
4	.048	−.024	.065	.085	.808
5	.526	−.066	.203	−.141	.168
6	.366	.608	.189	.150	−.054
7	.135	.604	.109	.242	−.034
8	.405	.338	.146	.255	−.138
9	.218	.625	−.051	−.072	.242
10	.050	.418	−.008	.303	.394
11	.756	.176	−.003	.231	.120
12	.784	.214	.152	.258	.046
13	.059	−.010	.696	.019	.174
14	.088	.130	.690	.057	.017
15	.159	.309	.574	.057	.215
16	−.055	.204	.666	.092	−.089
17	.120	.115	.676	−.021	.096
18	.077	.267	.166	.714	.012
19	.204	.389	−.022	.432	.052
20	.129	−.019	.050	.241	.538
21	.182	.765	.031	−.071	.204
22	.112	.600	.202	−.008	.014
23	−.009	.110	−.113	.060	.838

**Table 3:** Factors and items of sleep disturbance questionnaire in ACS patients.

Factors	Number	Items	Factor loading
First factor: sleep onset and continuity disorder	1	When I go to bed, it takes me a long time to fall asleep.	**0.807**
	2	Overall quality of sleep (no matter how long you slept)	**0.784**
	3	Awaken during my sleep time and have trouble falling asleep again	**0.762**
	4	Total sleep duration	**0.756**
	5	I wake up earlier than my preference time	**0.526**
	6	I am aggressive and irritable in the morning	**0.405**

The second factor: disorder in daytime functioning	7	I wake up suddenly from sleep with an unpleasant feeling of fear, anxiety, tension, or unhappiness	**0.765**
	8	I have a lot of nightmares in the sleep time	**0.713**
	9	I sometimes have unusual feelings in my legs at night, such as creeping, crawling, tingling burning, or itching sensations	**0.600**
	10	I wake up in the morning with feeling of fatigue and exhaustion	**0.608**
	11	I have headache in the morning after waking up	**0.604**
	12	I feel drowsy or sleepy during the day	**0.451**
	13	I have no enough concentration in the day because of sleepiness	**0.418**

Third factor: sleep disturbance caused by environmental factors	14	I can not sleep because of turned on lights	**0.696**
	15	I can not sleep because of environmental noise (such as cell phones, noise from medical instrument, crosstalk of nurses, and moan of other patients)	**0.690**
	16	I can not get into a comfortable position in bed (by bed or pillow) which causes sleep disturbance	**0.676**
	17	I can not sleep because of unpleasant ward's odor	**0.666**
	18	I wake up from sleep because of high increased or decreased temperature	**0.574**

Fourth factor: disorder caused by cardiac disease	19	I have cardiac problems in my sleep recently (chest pain, palpitation)	**0.714**
	20	I wake up from sleep because of chest pain	**0.432**

Fifth factor: respiratory disorder during sleep	21	I must sleep in special position (lying down on pillow, using two pillow for elevating head)	**0.838**
	22	I wake up from sleep because of snoring	**0.808**
	23	I wake up from sleep because of short of breath or chocking	**0.538**
